# Single-participant structural similarity matrices lead to greater accuracy in classification of participants than function in autism in MRI

**DOI:** 10.1186/s13229-021-00439-5

**Published:** 2021-05-10

**Authors:** Matthew J. Leming, Simon Baron-Cohen, John Suckling

**Affiliations:** 1 Department of Psychiatry, University of Cambridge, Robinson Way, Cambridge, Cambridgeshire CB2 0SZ UK; 2 Present Address: Center for Systems Biology, Massachusetts General Hospital, 149 13th Street, Boston, MA 02129 USA

**Keywords:** Autism, Deep learning, Functional connectivity, Structural similarity

## Abstract

**Background:**

Autism has previously been characterized by both structural and functional differences in brain connectivity. However, while the literature on single-subject derivations of functional connectivity is extensively developed, similar methods of structural connectivity or similarity derivation from T1 MRI are less studied.

**Methods:**

We introduce a technique of deriving symmetric similarity matrices from regional histograms of grey matter volumes estimated from T1-weighted MRIs. We then validated the technique by inputting the similarity matrices into a convolutional neural network (CNN) to classify between participants with autism and age-, motion-, and intracranial-volume-matched controls from six different databases (29,288 total connectomes, mean age = 30.72, range 0.42–78.00, including 1555 subjects with autism). We compared this method to similar classifications of the same participants using fMRI connectivity matrices as well as univariate estimates of grey matter volumes. We further applied graph-theoretical metrics on output class activation maps to identify areas of the matrices that the CNN preferentially used to make the classification, focusing particularly on hubs.

**Limitations:**

While this study used a large sample size, the majority of data was from a young age group; furthermore, to make a viable machine learning study, we treated autism, a highly heterogeneous condition, as a binary label. Thus, these results are not necessarily generalizable to all subtypes and age groups in autism.

**Results:**

Our models gave AUROCs of 0.7298 (69.71% accuracy) when classifying by only structural similarity, 0.6964 (67.72% accuracy) when classifying by only functional connectivity, and 0.7037 (66.43% accuracy) when classifying by univariate grey matter volumes. Combining structural similarity and functional connectivity gave an AUROC of 0.7354 (69.40% accuracy). Analysis of classification performance across age revealed the greatest accuracy in adolescents, in which most data were present. Graph analysis of class activation maps revealed no distinguishable network patterns for functional inputs, but did reveal localized differences between groups in bilateral Heschl’s gyrus and upper vermis for structural similarity.

**Conclusion:**

This study provides a simple means of feature extraction for inputting large numbers of structural MRIs into machine learning models. Our methods revealed a unique emphasis of the deep learning model on the structure of the bilateral Heschl’s gyrus when characterizing autism.

## Background

Voxel-based morphometry (VBM) [1] is a means of detecting structural differences in brain anatomy from T1-weighted MRI across groups. In VBM, images are registered to the same coordinate space and segmented into grey matter, white matter, and cerebrospinal fluid (CSF) volumes, before comparisons are made across voxels or groups of voxels using standard statistical tests. Due to its robustness and effectiveness, VBM has enjoyed significant popularity since it was first introduced [2, 3]. Structural covariance networks [4] correlate tissue volumes estimated by VBM in regions across groups of participants to describe relationships that are interpreted as measures of structural integrity or developmental coherence of the brain. These networks have been coupled with gene expressions [5] and correlated with disease-related alterations in brain topology [6], but their underlying neurophysiology is still an active area of study.

While there have been several cross-sectional findings of structural brain differences in autism [7–9], these have not been substantiated by a larger-scale analysis [10]. Indeed, characterizations of brain structure in autism have been inconsistent across studies of small sample sizes, although differences at different ages may explain some of this variation [11]; for instance, increased amygdala volumes have been reported in children with autism [12, 13], but not adults [8]. A meta-analysis of VBM studies in autism found disturbance of brain structure in the lateral occipital lobe, the pericentral region, the right medial temporal lobe, the basal ganglia, and proximate to the right parietal operculum [9]. Small-scale studies in children with autism have found altered structural covariance in areas associated with sensory, language, and social development. Altered structural covariance has been found between sensory networks, the cerebellum, and the amygdala in autism [14]. In children, [15] found that structural covariance indicated localized reductions within fronto-striatal and parietal networks and decreases in ventral and superior temporal grey matter, suggesting abnormalities in the anatomy and connectivity of limbic-striatal (i.e. social) brain system. Language ability correlated with cortical structure and covariance [16], and associations with language development are further supported by studies showing abnormal development of the Heschl’s gyrus [17], an area where functional activation has been associated with development of “inner speech” [18]. In adults with autism, structural covariance has shown decreased centrality in cortical volume networks [19].

Autism has been consistently associated with differences in brain function [20, 21]. Efforts to find differences in functional connectivity relative to neurotypical control groups have characterized autism as exhibiting under-connectivity, and thus greater segregation of functional areas [22–27]. Other studies, mostly of children and adolescents, found evidence of over-connectivity in specific areas of the brains of those with autism [28–33], locating hyperconnectivity to the posterior right temporo-parietal junction [29] and in striatal areas and the pons [30, 31]. [34] posited that autism is likely characterized by a mix of hyper- and hypo-connectivity traits.

### Machine learning

Machine learning has found multiple applications to the analysis of brain images in recent years, including pre-processing, segmentation, and diagnostics. Of great interest has been whole-brain phenotypic classification, in which MRI data of two or more phenotypes (such as sexes, or a diseased group and healthy controls) are trained and classified with a machine learning algorithm. Such studies most often include four steps: (1) selection of MRI modality and derived features that are sensitive to the problem at hand; (2) feature extraction, to reduce data dimensionality; (3) inputting features to train a machine learning model with the selected architecture; and (4) classification and interpretation.

MRI feature extraction is most often performed using techniques previously developed in image analysis, and the specific method is dependent on the selected modality and features. For instance, based on a large body of research and predictable dimensionality reduction [35, 36], it is common to use for classification functional connectivity matrices [37–39] representing correlations in time-series between pre-defined regions derived from blood oxygenation level-dependent (BOLD) sensitive fMRI. Likewise, to classify diffusion weighted images (DWI) it is common to use structural similarity matrices representing measures of white matter tracts traversing the brain between specific regions [40–42].

However, while there exists several consensus methods for deriving connectivities from fMRI [36, 43] and DWI [35] (though this is still an active area of research [44, 45]), analogous means of connectivity-based dimensionality reduction for T1-weighted structural MRI [46, 47] are not as widely used, even though this is the most common [48] modality available to study. One reason for the lack of common methodology is that reductions from three-dimensional data to network representations with meaningful physiological interpretation are more difficult to produce than reductions of four-dimensional data. In most existing feature extraction methods for T1-weighted MRI, extracted features are typically independent, univariate measures from regions of interest, such as cortical thickness and surface curvature. However, the lack of a connectivity metric leads not only to the loss of spatial encoding seen in network representations, but fewer features overall (i.e. for *N* ROIs, connectivities output $O(N^2)$ features while univariate measurements output *O*(*N*)), reducing effectiveness for machine learning.

For this study, we designed a similarity metric that reduced T1-weighted MRIs to a network representation without an a priori physiological interpretation, then applied it a dataset of autistic individuals and neurotypical controls. We applied this method to an extremely large dataset of participants with autism, representing a disorder for which structural characterization had proven difficult [49–52].

### Machine learning in autism

Previous machine learning studies of autism have achieved classification accuracies that widely varied depending on the modality used, sample size, data quality, selected methods, and diagnostic criteria. A recent study [53] of 106 high-risk infants between 6-12 months linked brain volume overgrowth to the emergence and severity of autism symptoms, using a deep learning algorithm capable of predicting autism with 81% specificity and 88% sensitivity using brain surface information. Another study by the same group [54] found that autism could be predicted in 59 6-month-old infants with 81.8% sensitivity using functional imaging. In the general population, efforts in single-participant classification of autism from MRI data have had mixed results [49, 55–60], with studies rarely exceeding 80% classification accuracy [34]. Again, however, this varies substantially by modality and which site data were collected [50]. In a recent study, Eill et al [61] performed a classification on individuals with autism and neurotypical controls using structural MRI, DWI, and fMRI data, finding that features derived from fMRI provided the highest accuracies with a support vector machine. They did, however, encounter the issue of fMRI feature extraction simply producing more variables than its structural counterparts, offering the machine learning model more information to work with, although attempts were made to mitigate this issue.

### Experiments

In the present study, we present a simple method of deriving structural similarity matrices from T1-weighted MRI. Our method compared the distributions of grey matter in pairs of parcellated areas of T1-weighted MRI. This metric acts as an indirect means of encoding relative size of grey matter volume while also being an effective means of dimensionality reduction that allows for T1-weighted MRIs to be encoded into a machine learning model. We describe our dataset, including acquisition and pre-processing methods. We validated these data using a machine learning model previously used in Leming and Suckling 2020 [62] and made comparisons to the classification accuracy using the corresponding fMRI connectivity matrices of the same participants, as well as lower-dimensional data consisting of grey matter volumes averaged within regions. Finally, we used the output class activation maps (CAMs), combined with graph theoretical techniques, to understand which parts of the brain the model focused on, and whether simple linear regression models could spot the same qualities in these data. We further describe a means of combining our structural similarity matrices with functional connectivity matrices in the same machine learning model to yield improved accuracy. Additionally, we show how the autism classification performance differed across age groups.

## Methods

### Dataset

We used a dataset comprised of 29,288 total instances each with a structural MRI and a functional MRI in both task-activated and task-absent (rest) conditions. (Note that instances were acquired from the same participant.) In total, 1555 data points were from participants with autism. These data were drawn from six different databases: Open fMRI, the UK BioBank, ABIDE I, ABIDE II, NDAR (minus ABCD), and ABCD (Table 1). Autism labels were collected in different ways depending on the format of the respective databases; for instance, ABIDE and BioBank had autism data already labelled, while NDAR and ABCD required keyword searches across different studies contributed by different groups; in such cases, unclear labels resulted in exclusion of data. Covariates of age, sex, and task were also compiled.

**Table 1:** Statistics for each dataset used

					Age				Sex		
Collection	Subj.	Conn.	Rest	Task	Min	Max	Mean	Stdev	F	M	Autism
ABCD	1049	5142	2296	2846	0.42	11.08	10.12	0.69	2474	2668	61
ABIDE	412	412	412	0	6.00	45.00	17.00	7.16	45	367	181
ABIDE II	682	717	717	0	5.22	55.00	14.39	7.39	169	548	350
BioBank	9791	9791	9791	0	40.00	70.00	55.00	7.51	5178	4613	4
NDAR	1050	7958	5531	2427	0.58	55.83	18.71	7.80	3816	4142	930
Open fMRI	1194	5268	820	4448	5.89	78.00	27.12	10.24	2346	2479	29
Total	14178	29288	19567	9721	0.42	78.00	30.72	–	14028	14817	1555

### Pre-processing and functional feature extraction

Functional data were pre-processed using SpeedyPP [63]. Data were first skull stripped using the Analysis of Functional Neuroimages (AFNI) toolbox. Motion was regressed from time series using wavelet despiking [63]. Data were then registered to the stereotaxic space of the Montreal Neurological Institute (MNI), after which they were overlaid on the 116-area AAL parcellation. Functional datasets with greater than 10% regional dropout, or either structural or functional datasets that otherwise failed the pre-processing stage, were excluded. The remaining datasets are presented in Table 1. 116 × 116 functional connectivity matrices were estimated using Pearson correlation on the averaged timeseries within a region.

#### GM volume and single-participant structural similarity matrices

**Fig. 1:**
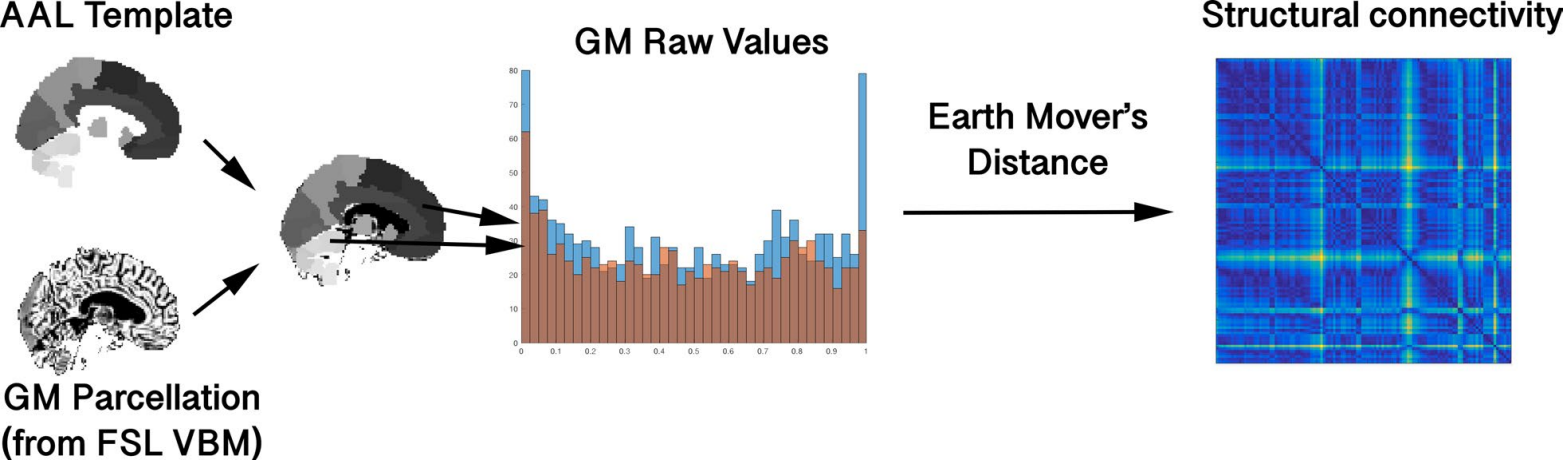
Illustration of the procedure used to estimate the structural similarity matrices used in the present study

To estimate grey matter volume distributions in each area in the AAL parcellation, structural MRI were first skull stripped using tools from AFNI, then registered to MNI space. Grey matter volumes were estimated using FSL VBM. We measured the similarity, *s* between two regions by comparing the distributions of nonzero voxel values within each AAL region (*u* and *v*), using the following equation:

1 $$\begin{aligned} s = \underset{\pi \in \Gamma (u,v)}{\inf } \int _{x,y \in \mathbb {R}\times \mathbb {R}}{|x - y|d\pi (x,y)} \end{aligned}$$

in which $\Gamma (u,v)$ is the set of distributions on $\mathbb {R} \times \mathbb {R}$ whose marginals are *u* and *v* [64]. This is simply the Wasserstein metric, which indicates the minimal amount of work necessary to transport one distribution to another. (In describing this metric, the two different distributions are often described as piles of dirt—hence its alternative name, “Earth-Mover’s distance”.) Prior inspection of these areas showed that the distribution of GM volume values followed no clear statistical distribution, and so this is an ideal metric as it nonparametically compares two statistical distributions, regardless of relative region sizes. A similar metric, the Kullback–Leibler divergence, has previously been used in brain morphology comparisons [46, 47], but this metric requires the estimation of a probability density function approximating grey matter distributions, because it is sensitive to histogram binning, whereas the Wasserstein distance is less so [65] and can be applied directly on discrete data. While this similarity metric does do away with spatial encoding and thus eliminates crucial information such as curvature, it acts as a comparison of the distributions of grey matter volumes between two areas in an easily understood way, and at a low computational cost. An illustration of this is shown in Fig. 1. Finally, univariate grey matter volumes were estimated by averaging nonzero voxels of GM volumes within each AAL region (effectively, the average of the distributions compared to find the structural similarity metric), producing a $116\times 1$ array of numbers. Because this needed to match the dimensionalities of the structural similarity and functional connectivity to be input into the CNN, these values were repeated to form a $116\times 116$ matrix.

### Comparison of structural similarity with functional connectivity

To determine whether functional connectivity and our novel structural similarity metric shared information in common, we correlated functional matrices from each instance with their corresponding structural matrices, in 10,000 random function/structural pairings (i.e. which were derived from the same subject) from across the whole dataset. We then compared these correlations with a null model estimated by correlating random pairings of functional and structural matrices (i.e. which were derived from different subjects). This was done by comparing the two sets of 10,000 R values with a t-test, indicating the amount of common information encoded by both structural similarity and functional connectivity.

### Machine learning model and training

We classified autistic individuals and neurotypical controls using, separately, structural similarity, grey matter volume, and functional connectivity measurements, as well as a model that combined structural and functional connectivities. We employed the model and training scheme described in Leming and Suckling 2020 [62] (submitted). This used an ensemble of 300 convolutional neural networks that each scrambled each symmetric $116\times 116$ input matrix into a randomly ordered $115\times 58$ rectangle, losing some spatial encoding information while avoiding biases in output class activation maps and maintaining the regularization effect of convolutions. Additionally, the use of CNNs allowed for the encoding of multiple connectivities (in this case, structural and functional) in different input channels, taking advantage of data structures that can alternatively encode RGB images. Matrices were then input to a CNN with 256 filters of shape $1\times 58\times 1$. This convolved $58\times 3$ random values of the matrix which was then fed into three dense layers, each with 64 hidden units, with batch normalization layers, rectified linear unit (ReLU), and 0.5 dropout between them. Finally, the data was binary classified through a softmax layer.

In building training, test, and validation sets for our models, a multivariate class balancing scheme was used [62]. For each autistic instance sampled in a given set, we sampled a neurotypical control participant of the same sex and data collection and with a statistically similar fMRI mean framewise displacement, intracranial volume, and age. The class balancing scheme further divided data into test, training, and validation sets for each model in the ensemble, ensuring participants with multiple functional connectomes were allocated to the same set. Each model was trained on an Adam optimizer for 100 epochs, with a copy of the model being saved at each epoch; the model that yielded the highest accuracy on the validation set was selected, and the prediction on the test set was the final accuracy reported.

For each classification task, we trained 300 independent CNNs, each with their own independently sampled and balanced training/test/validation set, then averaged their predictions for each data instance that fell within at least one test set. This effectively created an ensemble of CNNs, with each data instance having at least one CNN making a prediction. The final AUROC was derived by averaging predictions for each data instance that fell within a test set. Cross-contamination between training and test sets was prevented because the only predictions sampled were those of the independent model and its associated test set. Different aggregated AUROCs were recorded when the specific number of models in the ensemble varied; when adding models to the ensemble, the AUROC from the aggregated models increased in a predictable way. The AUROCs from between 20 and 300 models were fit to a logarithmic curve with a hard limit in order to predict the projected highest AUROC possible in the limit of a large number of models.

As a result of forced class balancing, each model in the ensemble used an independent subset of approximately 1600 instances, which was split between training, test, and validation sets at a ratio of 4:1:1. Because the multivariate class balancing scheme has stochastic elements and was run independently for each of the 300 models, there were small variations in the sizes of the final sets for each model, though variations were small (with a maximum size difference of about 50) and the size of the training set was overall consistent. For each model, different splits were used between training, test, and validation sets. As an effect of this balancing scheme, data from Open fMRI and the UK BioBank, having few participants overall with a diagnosis of autism, were included only infrequently, while data from ABIDE I and II, ABCD, and NDAR were frequently represented.

In total, four cross-sectional classification tasks were undertaken (Table 2), specifically: with structural similarity; with grey matter volumes; with functional encoding; and by combining structural and functional connectivities.

**Table 2:** Respective AUROCs and accuracies of ensemble models on different combinations of data

Modality	AUROC	Accuracy
Structural conn., function	0.7354	69.3980
Structural conn.	0.7298	69.7062
Function	0.6964	67.7180
Structure (GM vols)	0.7037	66.4228

### Class activation map analysis

Using the Guided Gradient Class Activation Map (Grad-CAM) algorithm [66], which displays areas of the input data most salient in classification, we measured the class activation of each data point in each model proposed, and then averaged these maps generating a $116\times 116$ CAM for both structural and functional connectivity, as well as a $116\times 1$ map for grey matter volume. We correlated the structural and functional CAMs to the measured effect size of differences between autism and neurotypical controls for our connectivity data, as a way to determine the similarity of CAMs to conventional statistics.

Next, we isolated hubs in the $116\times 116$ CAMs. To do so, we first measured the edge betweenness centrality of each edge in our CAMs. We then grouped these values into different communities by maximizing modularity of the edge betweenness values (Brain Networks Toolbox [67]). This procedure identified which hubs were most focused on by the classifier.

## Results

### Training

Accuracies and AUROCs on the test set are given in Table 2.

**Fig. 2:**
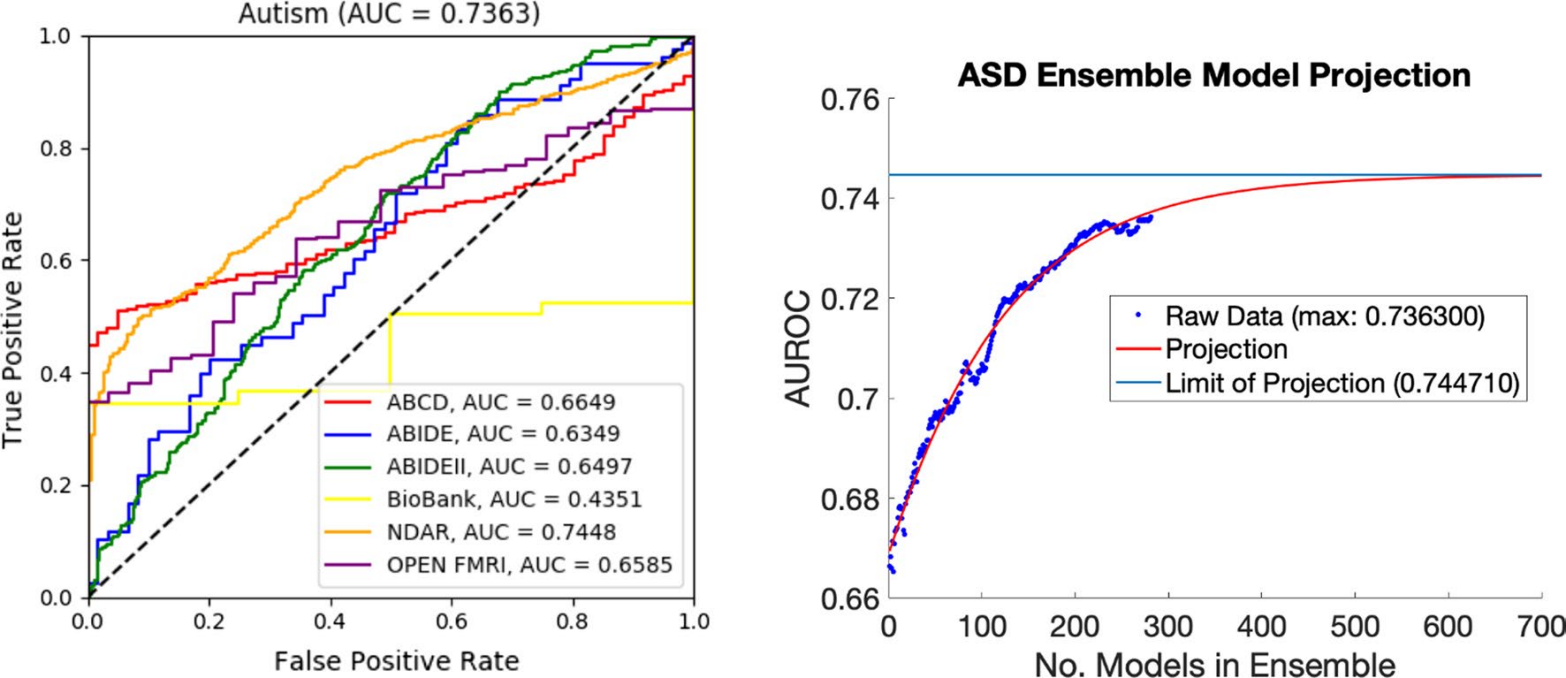
Left: overall AUROCs of each dataset included in the analysis for the structure/function ensemble. Right: projection of the limit of the structure/function/age ensemble models, given data for ensembles of one to 300 models. Adding independent models *ad infinitum* would result in a maximum predicted AUROC of 0.745

Classification resulted in a higher AUROC for structural than functional connectivities: 0.7298 and 0.6964, respectively. Classification on univariate grey matter volumes resulted in an AUROC of 0.7037, outperforming functional classification while underperforming structural similarity classification, although this might be expected considering its lower dimensionality. Combining structure and function resulted in an AUROC of 0.7354 (Fig. 2, left), with a projected upper limit of the AUROC of 0.745 (Fig. 2, right).

**Fig. 3:**
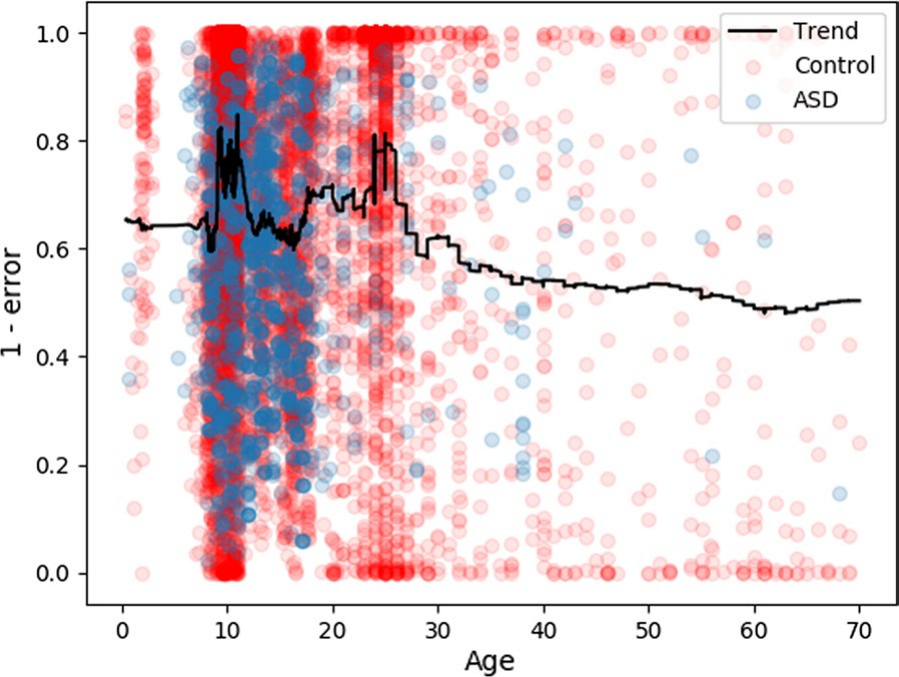
Relative classification error in the structure/function/age ensemble model, plotted against age. Each point in the graph represents the averaged classification error of the datapoint across each model in which it was included in the test set. Thus, more controls are represented which were each used individually in fewer models, while the autism datasets are fewer but were generally used in more models. This represents that accuracy was generally higher in the developmental age groups, likely because more data was present for those groups

Figure 3 shows the classification results across different age groups, reflecting the large disparities in age ranges present in the accumulated dataset, as well as the heightened model performance for those age ranges for which the most data was present. This reflects both the disparities in autism characterization across development as well as the likelihood of increased accuracy with more heterogeneous datasets.

### Class activation map analysis

**Fig. 4:**
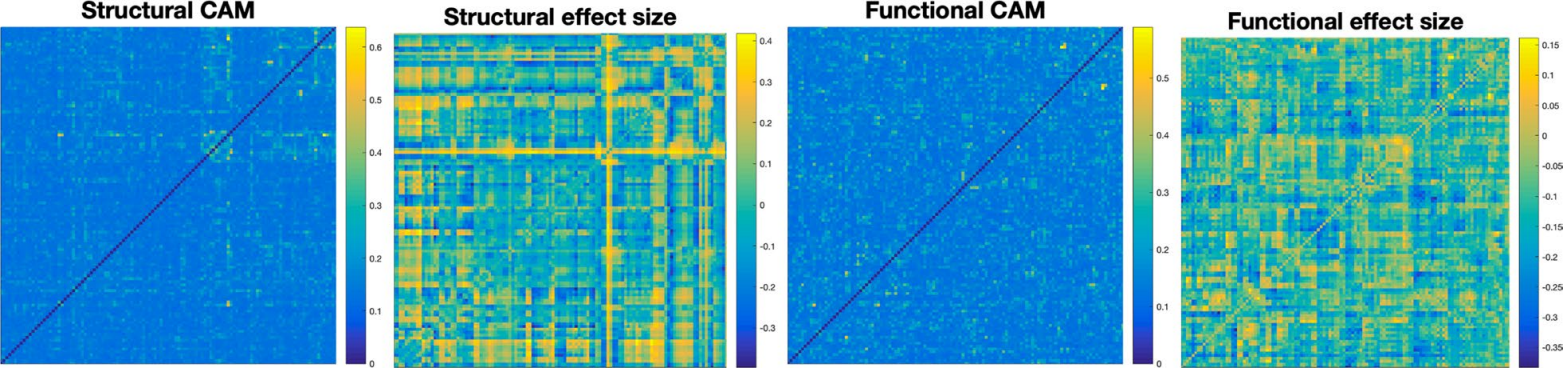
Comparison of the effect size of differences between raw matrix values between groups and the averaged class activation maps. Most of the edge differences passed a nonparametric statistical significance test. When comparing the CAM matrix and the effect size matrices using either linear or nonparametric correlation, neither had any statistically significant associations with one another

When comparing the output CAMs to their respective functional and structural effect sizes, no statistically significant correlation was observed, and thus, the machine learning model relied very little, if at all, on differences detectable by conventional statistics (Fig. 4).

**Fig. 5:**
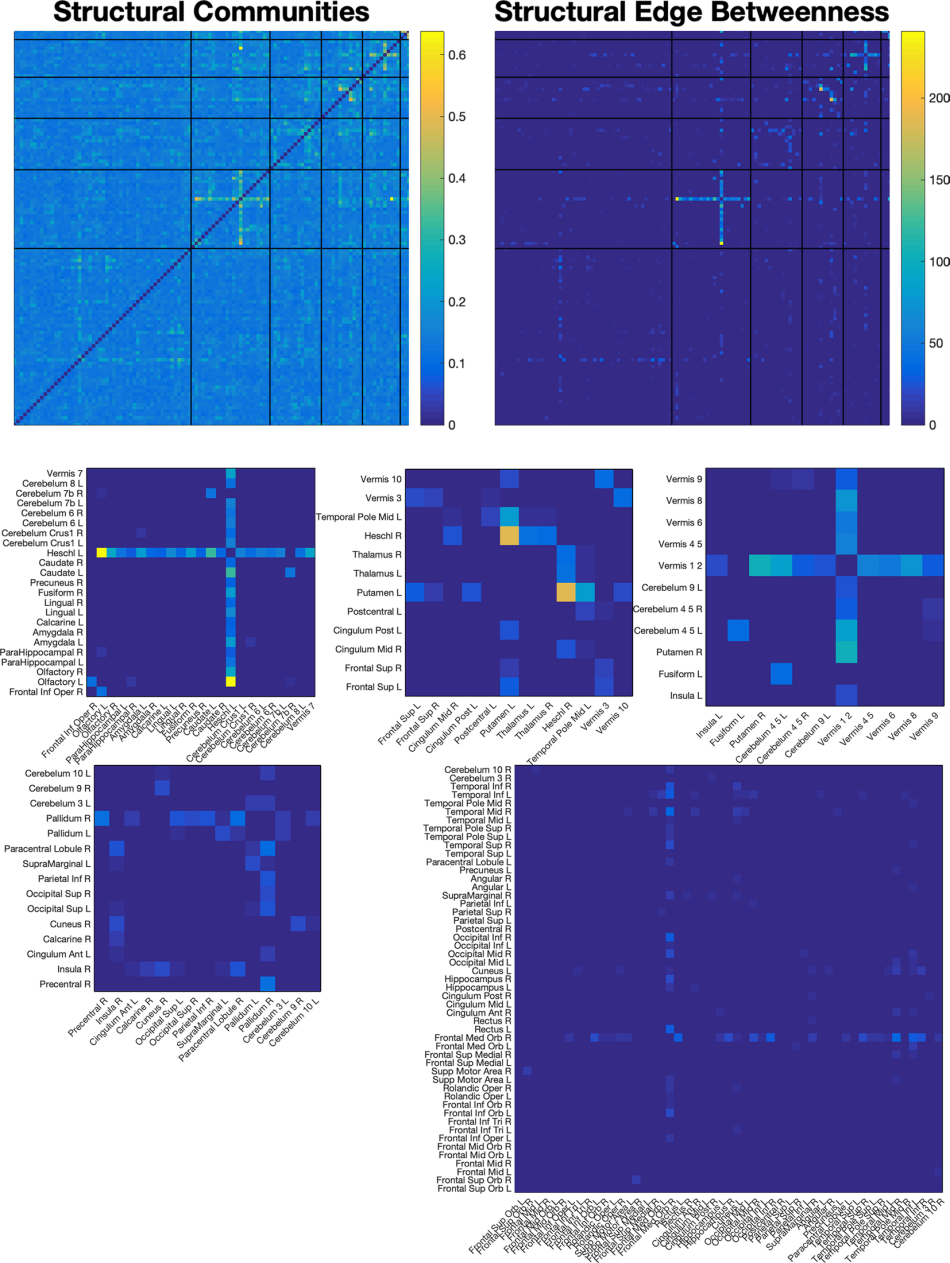
Structural hubs targeted by the structure/function/age encoding. Shown here are the class activation maps (upper left) as well as the edge betweenness centralities of the map (upper right), after it has been sorted into six different hubs via modularity maximization. The hubs, with labelled areas, are shown in the bottom half. (Middle) The three most distinct hubs revolve around the left Heschl’s gyrus; the right Heschl’s gyrus (and, to an extent, the left Putamen); and the upper vermis. The largest hub, in the bottom left, shows scattered-but-weak emphasis on connections to the right frontal medial orbital gyrus. These connections likely reflect the machine learning model’s use of comparisons of certain areas to others in order to assess the developmental difference of such areas in autism

**Fig. 6:**
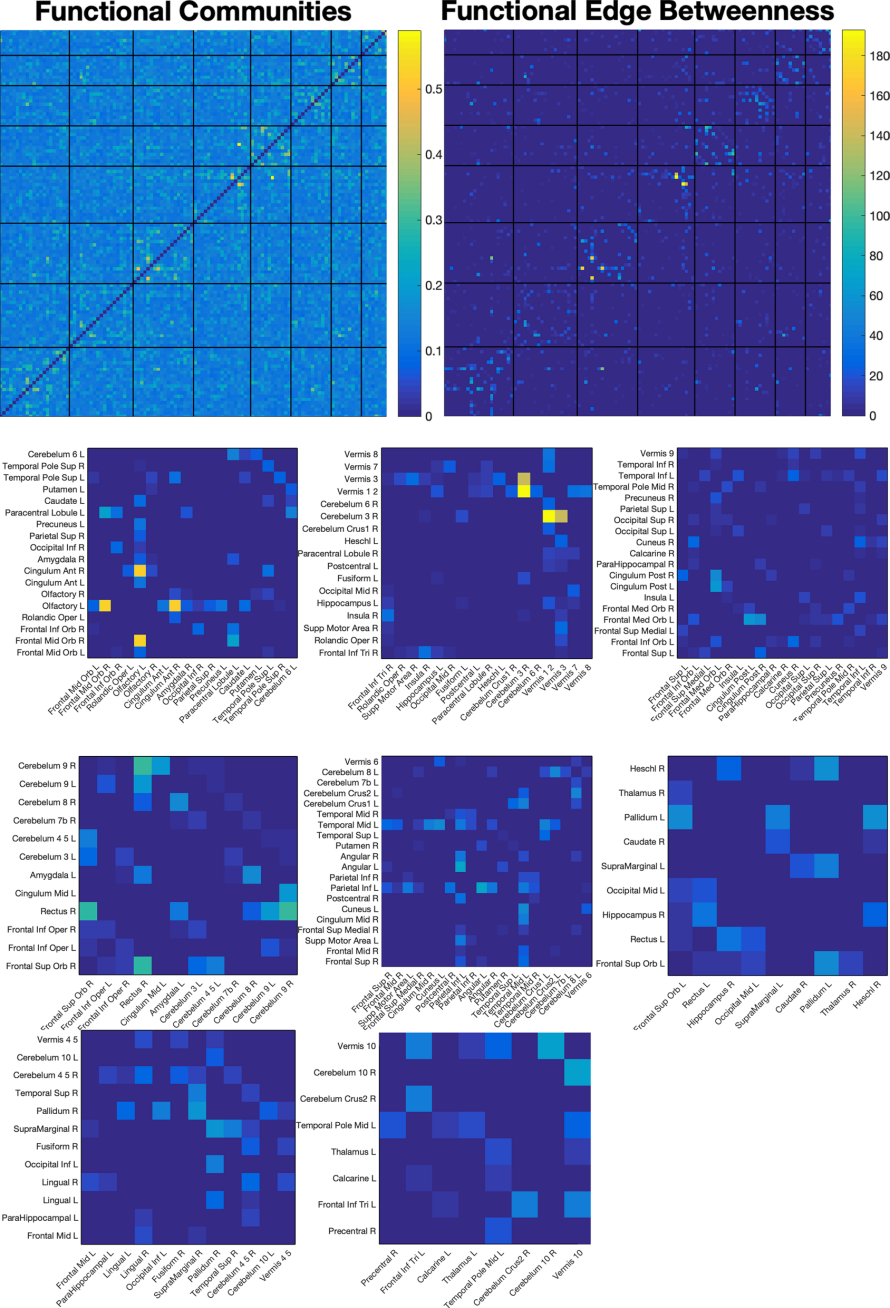
Averaged functional class activation maps and the associated edge betweenness centralities, when divided into communities via modularity maximization. Function does not show the same ultra-localized hubness within particular communities in the way that the structural results do, but emphasis is given to several individual connections throughout

CAMs for structural and functional connectivities, sorted by different detected communities after edge betweenness centrality was measured, are shown in Figs. 5 and 6. Structural CAMs showed five distinct groupings, each with distinct hubs that each centred on one or two localized areas, including the left and right Heschl’s gyrus, the upper vermis, the right frontal-medial orbital gyrus, the right pallidum, and the left putamen. The strongest activations were found in left Heshcl’s gyrus.

Localization was also found, though less distinctly, in functional hubs, notably the left inferior parietal lobe, the left middle temporal lobe, the left olfactory bulb, and the upper vermis. However, focus on particular hubs was not a distinctive feature.

**Fig. 7:**
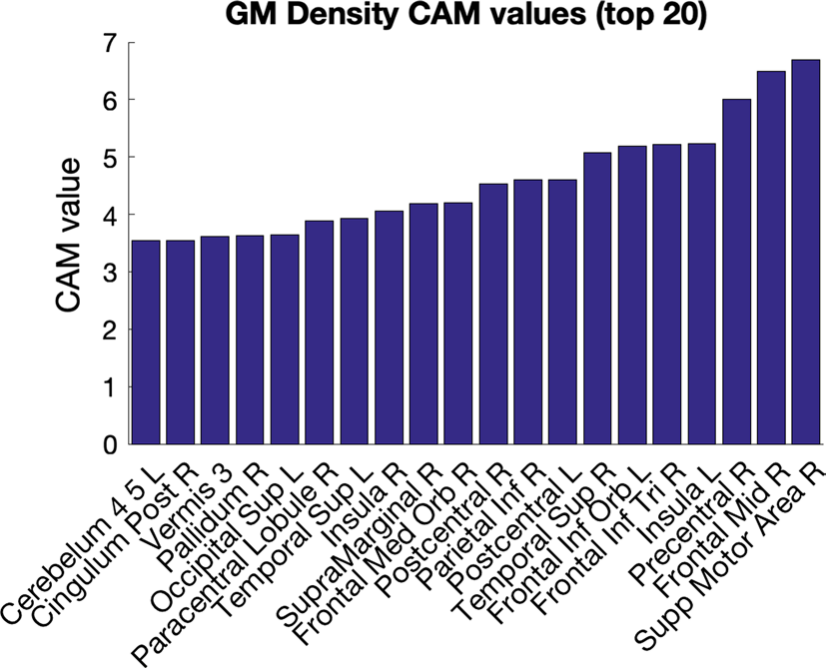
Top class activation map value results for the 116-area grey matter density classification, showing the areas most focused on in that classification task. The minimum CAM value (not shown) was 1.3622

CAMs for grey matter volumes are shown in Fig. 7. These results had very little in common with the structural similarity results, with the strongest five activated areas in the right supplementary motor area, the right middle frontal lobe, the right precentral sulcus, the left insula, and the inferior frontal gyrus triangularis.

## Discussion

This study proposed a new feature extraction method for inputting structural MRIs into a network-based machine learning model, as well as applicable analysis methods to detect areas of that were particularly involved in determining the classification. Estimating single-participant structural similarity matrices from T1-weighted images without supplementary modalities such as DWI or fMRI is uncommon, and research in this area is ongoing [46, 47, 68]. In structural covariance, VBM data are used to produce inter-regional relationships at a group level, but this is inapplicable at a single-participant level, which is necessary to make structural MRIs applicable to machine learning models. The proposed method provides a means of doing so.

In developing this method, other means of estimating single-participant connectivity matrices from T1-weighted MRI were considered, such as estimating the correlation between different univariate measurements (cortical thickness, curvatures, and so on) of the structural image [44, 45], but this was too computationally intensive for a large dataset. Another method was investigated that involved finding the difference between group structural covariance matrices with and without a certain participant. While classifications on these matrices were successful, the matrices themselves varied to such an extent that the output CAMs were inconsistent; rather than describing physiological features, these matrices described deviations of individuals’ physiological features, and as a result the matrices themselves had no consistent features that could be locally focused on by our DL model. The use of the Kullback–Leibler metric used in Kong et al [46] was also considered, but for the reasons described above, the Wasserstein metric was decided upon because of its simplicity and effectiveness in classification.

In the univariate grey matter volume results, the CAMs highlighted the right supplementary motor area, right mid frontal lobe, right precentral sulcus, left insula, right frontal inferior triangularis, left frontal inferior orbital lobe, and the right superior temporal lobe. (The top 20 areas are shown in Fig. 7.) Comparing the CAM emphasis of the grey matter volumes to the meta-analysis of autism VBM studies in [9], which found six areas with consistently altered grey matter volumes, some similarities can be seen, notably in the right superior temporal lobe where grey and white matter volume differences in the right medial temporal lobe and the left post central gyrus.

Functional analysis did not reveal a pattern of local hubness characterizing structural connectivity differences, but rather focused on specific connections. However, a number of general functional communities were identified (Fig. 6). Meta-analyses of studies in functional connectivity differences associated with autism have not found consistent differences in the brain, but rather in network-wide measures [34]. The lack of hub emphasis in functional results may be additional evidence of network-wide, rather than localized differences between autism and neurotypical control groups seen in other recent findings [69].

In structural similarity, three definitive hubs were identified: left Heschl’s gyrus, right Heschl’s gyrus, and the upper vermis. The right pallidum and fronto-medial orbital region also showed relatively strong local hubs, though to a lesser degree. Emphasis of the Heschl’s gyrus is in agreement with recent studies in developmental autism, having been implicated previously as an area that develops atypically in autistic children [17]. Function of the area has been associated with development of “inner speech” [18], indicating a difference in development of language capabilities. Our findings differ in that they found this emphasis in *structure* and not *function*, but this may be reflective of the lower variability of differences across a single area in the development of grey matter as opposed to function, which likely varies far more across participants, and age groups. The cerebellum, meanwhile, has consistently been cited as an area of difference between individuals with autism and neurotypical controls during development [11], as well as an area of difference in structural covariance associated with autism [14].

The structural similarity CAMs resulting from this study revealed an emphasis on a number of distinct and localized areas, and these areas were clarified by use of an edge centrality measurement combined with modularity maximization to isolate hubs. The edge betweenness step was added by necessity to place extreme emphasis on a smaller number of more central edges, and only then could modularity maximization isolate hubs in a meaningful way (see Fig. 5).

**Fig. 8:**
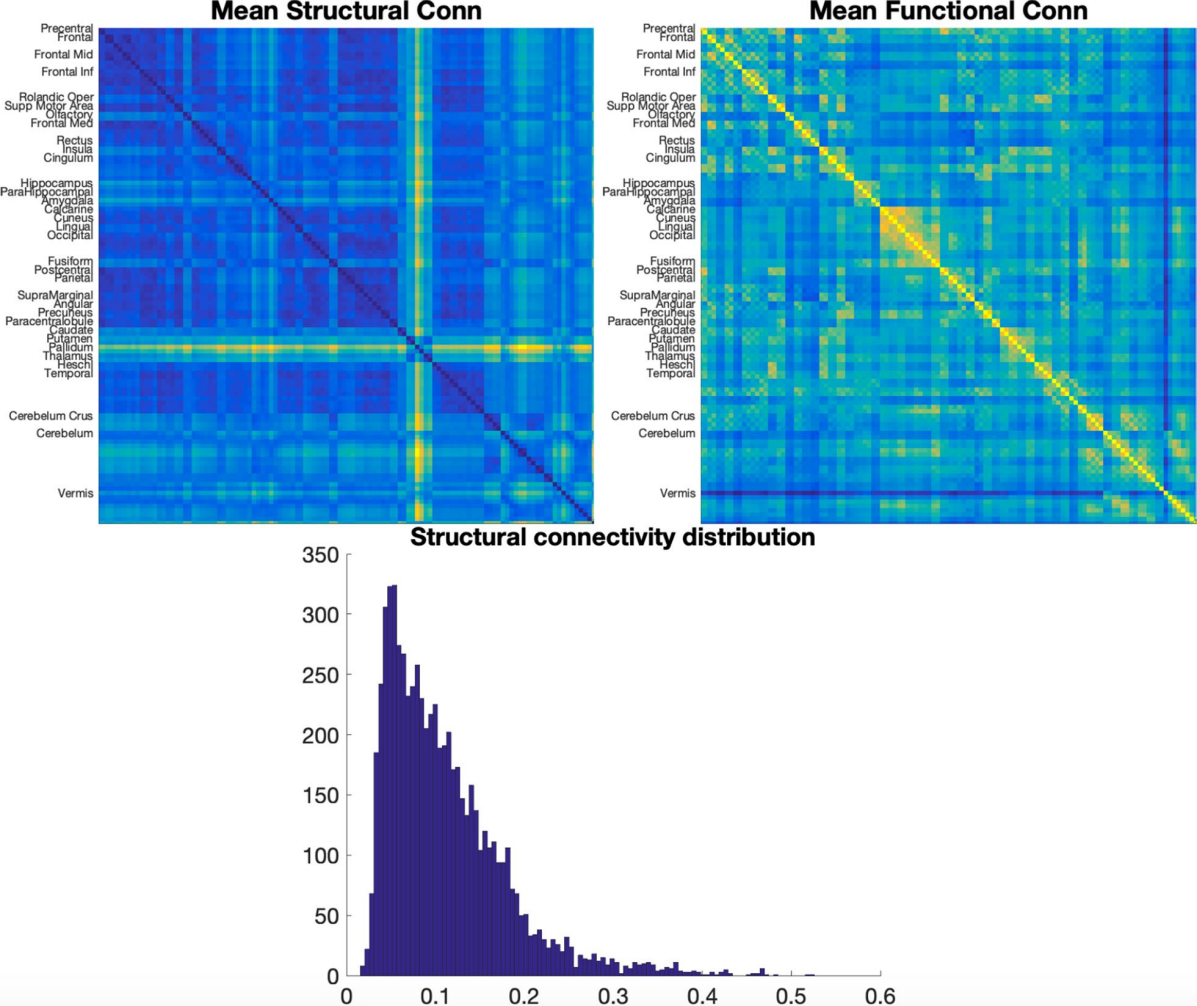
Average structural (left) and functional (right) connectivity matrices. The distribution of values of the structural similarity metric is also shown

The structural similarity method’s efficacy with the machine learning model suggests that it encoded practically useful information about brain structure, but the interpretation of what these structural hubs indicate physiologically is more complicated. While some correlation is present (Fig. 8) the functional connectivity and structural similarities show largely different patterns. Furthermore, considering that the method used to estimate structural similarity was a similarity metric, the emphasis on these hubs was less likely an indication that they were centres of a physiological brain network characterizing autism. Because the strength of connections was a comparison of grey matter distributions, it is more likely that connections to the identified hubs were used by the machine learning algorithm as a proxy for detecting subtle changes in the morphology of grey matter within those specific regions. Edges connecting to these structural hubs were probably an indirect indication of differences in grey matter between two areas, and the individual connections themselves would not indicate any special physiological relationship, but rather a comparison of grey matter volume. However, this still means that the hubs themselves were important in characterizing autism, and, as this work has shown, it has clear utility in the context of machine learning. This structural similarity metric may simply be viewed as a way of encoding relative spatial information about the morphology of individual areas of the brain.

The univariate grey matter volume results further complicate interpretation because areas different from the structural similarity results were emphasized by the CAMs, even though both univariate grey matter volumes and structural similarities were derived from the same imaging data. This brings up three key points. First, the method of encoding data is important because it presents different types of information to the machine learning model. Structural differences in autism (and likely other phenotypic differences) may vary in different ways that are only apparent under specific methods of encoding, and thus, the model may have focused on different areas, depending on which method of encoding was performed. This is important for both interpreting the results in the context of a specific machine learning task and understanding the underlying physiological implications. Second, the emphases presented by Grad-CAM were *relative*; that is, in analysing the distribution of Grad-CAM values, we saw that the model took all areas into account (Fig. 7), although with highest focus on the few areas that seemed to hold more influence in the final classification task. This does not, however, mean that other areas were ignored entirely. Third, because of the higher dimensionality of structural similarities over grey matter values, it may be the case that the machine learning model assumed information about grey matter volumes from a small number of edges, while information about differences in morphology of other areas (e.g. the left and right Heschl’s gyrus), which were not present in the univariate feature extractions, required emphasis by a greater number of edges; this may be crucial to understanding differences in autism generally, or it may have simply helped the model increase AUROC by a margin of 0.0891 between the univariate and connectivity classification tasks. Stated informally, differences in morphology detected by the structural similarity matrices were more subtle, and so they required the emphasis of a larger number of edges.

The results in Fig. 3 show that autism classification, even when structure and function are considered, generalizes poorly across large age groups. This supports the findings in recent longitudinal studies of autism [70–72], which found high inter-individual variability in brain volume growth trajectories in autism. This suggests that autism is highly variable in its development and that information about one age group with autism would not necessarily inform predictions of another age group. To properly generalize these findings in the context of this study, more data from older age groups would be needed.

The differences between ensemble AUROCs are small in some cases, but these represent the convergent AUROC of 300 individual models rather than a single trained model, which would be more subject to classification error. Evidence for this is shown in Fig. 3, which shows a predictable convergence of test set accuracy as more models are added to an ensemble. This indicates that the observed AUROCs are reproducible in the context of this study, though further analyses are necessary to form strong conclusions about the neurobiology of autism.

Because of this, we can be sure that the AUROCs represented for each set of features are reproducible, and while the advantages of integrating multiple sets of features does lead to increased performance, this increase is only slight. This may indicate an upper limit on possible test set accuracy given the features represented in the data; in other words, the presented data may simply not have patterns present that can generally indicate differences between autism and controls, and so integrating more features into a deep learning model has diminishing returns.

It is notable that none of the classification accuracies presented in this paper approached the success required for a clinical diagnosis, which would need to consistently exceed 95% accuracy on a substantially large dataset. A likely reason for the comparatively low accuracy in this study specifically is the large dataset size, which, in the context of whole-brain MRI classification, has previously been associated with a drop in accuracy [50, 73], as well as the possible lack of neurobiological signatures that would be able to sufficiently distinguish autist subjects from typical controls. Nonetheless, deep learning models are useful in these contexts both as statistical models in and of themselves to study autism, and as building blocks to approach clinical-quality accuracy in the future.

Finally, we combined our structural similarity metric with functional connectivity raising the final AUROC. This shows that our method does not have to be considered as a replacement for any previous methods, but may be used in combination with them in order to make single-participant classifications more effective.

## Limitations

This study has several limitations. While we used a large sample size, most data were drawn from a relatively narrow age group, and so generalizations of the findings to adult autism may be inappropriate. Additionally, with the extreme heterogeneity of autism and the different sites and studies the data was drawn from, as well as varying reliability of autism labels depending on diagnostic criteria in different sites, a binary machine learning task of the type described here may be ill-formed. Nonetheless, given lack of richer information within the data, options for design of the machine learning problem are limited. Finally, insights into areas of the brain deemed salient by the deep learning visualization methods may be specific to the methods used to derive the brain connectome, or to a specific subgroup in the data, and further studies are required to assess the generalizability of these findings.

## Conclusion

The present study offers a means of encoding T1-weighted MRI for use in network-based machine learning models, and with a machine learning classification task we have demonstrated an increase in accuracy in classifying autistic individuals when compared with both functional connectivities and classification of univariate grey matter volumes. Furthermore, we presented methods of identified areas emphasized by the machine learning model, demonstrating the importance of data encoding and highlighting complications with interpreting results when the feature extractions have no specific physiological interpretation. While this trade-off, interpretability for higher accuracy, will likely continue to be an issue in machine learning with scientific data, the effects of data encoding on accuracy point towards feature extraction methods as a future direction of investigation.

## Data Availability

All data analysed in this article is public. Code for analysis is available on Github.

## Abbreviations

ABCD: Adolescent brain cognitive development
ABIDE: Autism brain imaging data exchange
AFNI: Analysis of functional neuroimages
ASD: Autism spectrum disorder
BOLD: Blood oxygen level-dependent
CAM: Class activation map
CNN: Convolutional neural network
CSF: Cerebrospinal fluid
DWI: Diffusion-weighted imaging
fMRI: Functional magnetic resonance imaging
Grad-CAM: Gradient class activation map
MNI: Montreal neurological institute
MRI: Magnetic resonance imaging
NDAR: National database for ASD research
ReLU: Rectified linear unit
ROI: Region of interest
VBM: Voxel-based morphometry
